# Effect of Dietary Fiber and Metabolites on Mast Cell Activation and Mast Cell-Associated Diseases

**DOI:** 10.3389/fimmu.2018.01067

**Published:** 2018-05-29

**Authors:** Jelle Folkerts, Ralph Stadhouders, Frank A. Redegeld, See-Ying Tam, Rudi W. Hendriks, Stephen J. Galli, Marcus Maurer

**Affiliations:** ^1^Department of Pulmonary Medicine, Erasmus MC, Rotterdam, Netherlands; ^2^Division of Pharmacology, Department of Pharmaceutical Sciences, Faculty of Science, Utrecht University, Utrecht, Netherlands; ^3^Department of Pathology, Stanford University School of Medicine, Stanford, CA, United States; ^4^Department of Dermatology and Allergy, Charité – Universitätsmedizin Berlin, Berlin, Germany; ^5^Department of Cell Biology, Erasmus MC, Rotterdam, Netherlands

**Keywords:** mast cells, allergy, asthma, dietary fiber, short-chain fatty acids

## Abstract

Many mast cell-associated diseases, including allergies and asthma, have seen a strong increase in prevalence during the past decades, especially in Western(ized) countries. It has been suggested that a Western diet may contribute to the prevalence and manifestation of allergies and asthma through reduced intake of dietary fiber and the subsequent production of their metabolites. Indeed, dietary fiber and its metabolites have been shown to positively influence the development of immune disorders *via* changes in microbiota composition and the regulation of B- and T-cell activation. However, the effects of these dietary components on the activation of mast cells, key effector cells of the inflammatory response in allergies and asthma, remain poorly characterized. Due to their location in the gut and vascularized tissues, mast cells are exposed to high concentrations of dietary fiber and/or its metabolites. Here, we provide a focused overview of current findings regarding the direct effects of dietary fiber and its various metabolites on the regulation of mast cell activity and the pathophysiology of mast cell-associated diseases.

## Introduction

Western diets are characterized by the consumption of energy-rich foods and beverages, typically high in fat, sugar, and salt but low in dietary fiber. These diets have been shown to affect the immune system and are thought to promote the development of a variety of immune disorders, including allergies and asthma (1, 2). Indeed, the past decades have seen a strong increase in the prevalence of allergic diseases in developed countries with a Western lifestyle, parallel to a reduced consumption of dietary fiber (3). Immune disorders that are thought to be most affected by the change in dietary fiber intake include asthma, eczema, hay fever, and food allergy (4, 5). A recent example comes from the reunification of East and West Germany, after which a significant increase in the prevalence of childhood hay fever and allergic sensitization was reported in East Germany, following exposure to several years of Western living conditions (6). Intriguingly, disorders that are most affected by reduced fiber consumption are often strongly linked to mast cell activity (3). However, most of the research regarding the effects of Western diet on such disorders has primarily focused on disease initiation *via* B and T-cell activation, rather than disease manifestation itself. Hence, the effects of dietary fiber and its metabolites on mast cells and other effector cells of allergy and asthma remain poorly understood.

Dietary fiber consists of non-digestible carbohydrates sourced from plant polysaccharides and plant or human milk-derived oligosaccharides. They are resistant to enzymatic and chemical digestion until they reach the large intestine, where they are fermented to short-chain fatty acids (SCFAs) and other metabolites by gut bacteria (7). Mammals, including humans, are deficient in the enzymes required to degrade the bulk of polysaccharides and resistant oligosaccharides, as illustrated by decreased amounts of SCFAs in germ-free mice, which lack bacteria in the gut (8). A high-fat/low-fiber diet is accompanied by an increase in the Firmicutes/Bacteroidetes species ratio, which is associated with different disease types, including obesity (9). In contrast, a high-fiber diet leads to an increased Bacteroidetes to Firmicutes ratio and elevated concentrations of SCFAs (10, 11). The potential role of gut microbiota in allergic diseases and asthma has been well documented and extensively reviewed (12–14). Here, we will provide a focused overview of the current findings regarding the direct effects of dietary fiber and its metabolites on the regulation of mast cell activity and the pathophysiology of mast cell-associated diseases.

## Dietary Fiber—its Source, Metabolism, and Biological Impact

In contrast to starch and starch-like polysaccharides that are easily hydrolyzed by enzymatic reactions and absorbed in the small intestine, dietary fiber is neither digested nor absorbed until after bacterial fermentation in the large intestine. Defining and categorizing dietary fiber is complex and challenging due to a large variety in their nutritional, functional, and chemical properties. The American Association of Cereal Chemists defines dietary fiber as “carbohydrate polymers with more than a three-degree polymerization, which are neither digested nor absorbed in the small intestine” (15) (Table 1). However, this definition incorporates a great variety of fiber. In the field of (allergic) inflammation and immunology non-starch polysaccharides (mainly found in vegetables, fruits, and cereals), oligosaccharides (primarily found in plants, beans, and human milk), together with specific analogous carbohydrates, such as resistant starch, recently received particular attention. Therefore, we will focus on the effects of these dietary fiber components and its metabolites. The role of other dietary fiber components and metabolites on the immune system has been reviewed elsewhere (16–18).

**Table 1:** Constituents of dietary fiber.^a^

Non-starch polysaccharides and resistant oligosaccharides
Cellulose
Hemicellulose
Arabinoxylans
Arabinogalactans
Polyfructoses
Inulin
Oligofructans [fructo-oligosaccharides (FOS)]
Galacto-oligosaccharides (GOS)
Gums
Mucilages
Pectins

**Analogous carbohydrates**

Indigestible dextrins
Resistant maltodextrins (from corn and other sources)
Resistant potato dextrins
Synthesized carbohydrate compounds
Polydextrose
Methyl cellulose
Hydroxypropylmethyl cellulose
Indigestible (“resistant”) starches

**Lignin-substances associated with the non-starch polysaccharide and lignin complex in plants**

Waxes
Phytate
Cutin
Saponins
Suberin
Tannins

*^a^Dietary fiber components as defined by the American Association of Cereal Chemists. Adopted from “The Definition of Dietary Fiber” (15)*.

Both polysaccharides and resistant oligosaccharides are potent substrates for the production of SCFAs. SCFAs are known to exert their biological effects through activation of membrane receptors GPR41, GPR43, and GPR103 (19–21), as well as the peroxisome proliferator-activated receptor (PPAR) nuclear receptor family (22). PPARs function as transcription factors, following the formation of a heterodimer with the retinoid X receptor, and regulate the expression of genes that are involved in both metabolism and immunity (23). SCFAs also possess the capacity to inhibit histone deacetylase (HDAC) activity (24–26), which is known to regulate gene expression, as well as the acetylation of non-histone proteins, including transcription factors (27). Acetate, propionate, and butyrate are the most extensively described SCFAs and are found in the intestinal tract at a molar ratio of 60:20:20, respectively (28). Importantly, SCFAs are not restricted to the intestinal tract, but can disseminate systemically and are detectable in the blood (29).

## Effects of Dietary Fiber and its Metabolites on Mast Cell Function

**Figure 1:**
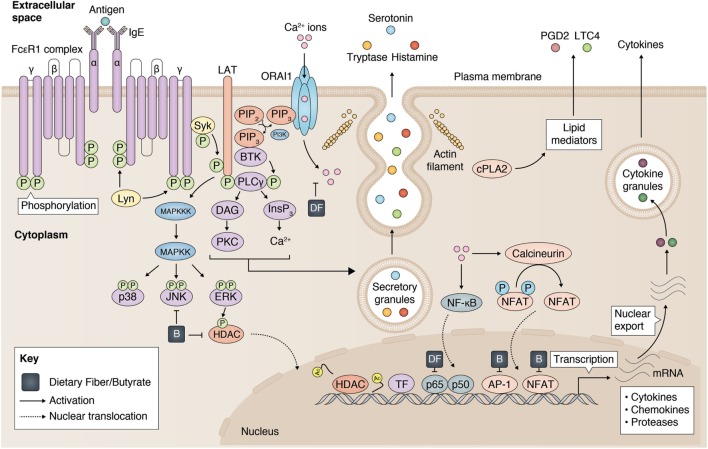
Inhibition of mast cell activation by dietary fiber and butyrate. Mast cell activation is modulated by dietary fiber and butyrate *via* (1) a reduced calcium entry, (2) inhibition on JKN/p38 phosphorylation, (3) reduced histone deacetylase (HDAC) activity, and (4) regulation of p65, AP-1, NFAT activity. Partly adopted from Cildir et al. (40).

Mast cells play a central role in initiating and maintaining inflammation, particularly in allergies and asthma (30). Although mast cells can be found in all well vascularized tissues, they are predominantly found in tissues where our body makes contact with the outside world (31). These tissues include the gastrointestinal tract (GI), the upper and lower airways, and the skin. The local or systemic presence of plasma-cell derived immunoglobulin E (IgE) can prime mast cells *via* the high affinity receptor FcεRI (30). Re-exposure to a specific allergen induces FcεRI aggregation on the plasma membrane, which can trigger mast cell degranulation within minutes, releasing numerous inflammatory mediators, such as serine proteases (tryptase and chymase) and histamine (32). Subsequently, downstream signals initiate the transcription and secretion of many pro-inflammatory cytokines, including TNF (33, 34) and IL-6 (35). Although the complete sequence of events that leads up to mast cell activation is not fully understood, it is known that aggregation of FcεRI results in the phosphorylation of the linker for activation of T cells (LAT) adaptor molecule in a LYN and SYK (spleen tyrosine kinase) dependent manner (36) (Figure 1). This sequence of signaling events subsequently causes activation of PLCγ and protein kinase C (PKC), which increases the mobilization of calcium (Ca^2+^) to initiate mast cell degranulation (36). On the other hand, *de novo* synthesis of eicosanoids (such as leukotrienes and prostaglandins) and transcriptional activation of cytokine genes (including TNF and IL-6) are induced by the activation of the mitogen-activated protein kinase (MAPK) pathway. Activation of the MAPK proteins extracellular signal-regulated kinase 1 (ERK1) and ERK2 are known to be regulated by RAS/RAF complex and play a major role in cell differentiation and proliferation (37). MAPK kinases (MAPKKs) and the MAPKK kinases (MAPKKKs) that mediate activation of p38 and c-Jun N-terminal kinase (JNK) in mast cells are less well-defined (38, 39), but are generally associated with apoptosis and inflammation.

Mast cell activation can also be induced by many IgE-independent stimuli, such as neurotransmitter substance P or complement anaphylatoxins (e.g., C3a and C5a) (22). Interestingly, different mast cell activators can induce cell activation using different types of degranulation strategies (41). However, the involvement of the MAPK signaling pathway in mast cell activation seems to be preserved regardless of the nature of the stimulus (38, 42, 43). Importantly, both MAPKs as well as a Ca^2+^ influx strongly regulate transcription of cytokines *via* transcription factors, such as NF-κB, NFAT, and AP-1 (40). For example, the nuclear localization (and, therefore, effective DNA binding) of NFAT transcription factors is controlled by an interplay of Ca^2+^ signaling with other signaling pathways (e.g., MAPK) (44). Second, various transcription factors normally reside in the cytosol, and make their passage into the nucleus through the nuclear envelope *via* nuclear pore complexes (NPC) to reach the chromatin. Increases in cellular Ca^2+^ (45) and MAPK-induced phosphorylation of certain compartments of the NPC (e.g., nucleoporins) (37) can regulate the nuclear translocation of these transcription factors. Finally, nuclear MAPKs have been shown to directly regulate chromatin remodeling, thereby modulating DNA accessibility (37). Erk1/2 was reported to interact with and phosphorylate HDAC4 *in vitro* (46). Additionally, activation of the ERK1/2 pathway induces the translocation of HDAC4 into the nucleus, thereby potentially regulating chromatin state.

Although the effects of dietary fiber have been investigated extensively in disease that are associated with mast cell activation, there have been few published reports demonstrating its effects on mast cell activation and function. A recent study has shown that Angelica polysaccharide (AP), a major source of fiber from the medicinal herb Angelica, can inhibit mast cell histamine release in a dose-dependent fashion as well as mediator synthesis *via* reduced Ca^2+^ entry, p38 phosphorylation, and NF-κB p65 expression (47). Additional studies have supported these findings by demonstrating that both polysaccharides (48, 49) and oligosaccharides (50) can directly inhibit mast cell degranulation and pro-inflammatory cytokine production *in vitro*.

In contrast, the effects of microbial metabolites, SCFAs, on mast cell function have been studied in more detail. In an early report, Galli et al. showed that MC/9 mouse mast cells exhibited decreased cell proliferation and increased histamine content and cytoplasmic granules when exposed to butyrate *in vitro* (51). Recently, Diakos and colleagues (52) were the first to report that butyrate can inhibit mast cell degranulation and TNF production. Butyrate administration significantly inhibited the phosphorylation of JNK, but not Erk1/2 and p38, thereby potentially regulating mast cell activation and TNF production. Production of TNF following IgE-dependent mast cell stimulation is known to be strongly regulated at the level of gene transcription (53, 54). Therefore, the authors assessed the binding of different transcription factors in the *TNF* promoter region and found that butyrate significantly inhibited nuclear binding of AP-1 and NF-AT.

The effects of butyrate on mast cell degranulation were more recently confirmed by Wang et al. (55), who demonstrated that sodium butyrate pretreatment reduced the percentage of degranulated mast cells, decreased mast cell mediator content, and lowered mRNA expression of pro-inflammatory cytokines in jejunal mucosa preparations isolated from weaned pigs. In line with a previous report (52), this study also found that butyrate affected the MAPK signaling pathway by inhibiting the phosphorylation of JNK, but not of Erk1/2 and p38.

However, a recent report by Zhang et al. showed that butyrate inhibited FcεRI-dependent release of TNF and IL-6 from mouse bone marrow-derived mast cells (BMMCs) without affecting degranulation (56). Moreover, butyrate pretreatment decreased c-Kit expression of mouse mastocytoma P815 cells and suppressed their cell proliferation by inducing cell cycle arrest and apoptosis. Furthermore, the transcriptional activity of TNF and IL-6 in antigen-stimulated BMMCs, was inhibited by butyrate as well as trichostatin A (TSA), a known HDAC inhibitor (56). Paradoxically, the authors also showed that butyrate-induced inhibition of HDAC activity leads to increased histone (H3K9) acetylation at the promotor regions of the *TNF* and *IL6* genes (56)—a chromatin state strongly linked to gene activation (57, 58). As the increased histone acetylation of the chromatin is unlikely to explain the decreased cytokine transcription, butyrate exposure appears to suppress inflammatory cytokine expression *via* chromatin acetylation of upstream regulator genes (i.e., repressors of TNF and IL-6 transcription) or non-histone protein acetylation. Although less well studied than histone acetylation, many non-histone proteins (including transcription factors) are subjected to acetylation, impacting numerous nuclear and cytoplasmic processes (59, 60). Butyrate and other dietary components with HDAC inhibiting activity have been reported to be key facilitators in non-histone protein acetylation (61). To explain the contradictory effects found on acetylation of the histones, Zhang et al. also assessed butyrate-induced acetylation of α-tubulin in mast cells (56), which is controlled by the tubulin-associated HDAC6 (62). Butyrate inhibits most of the classic HDACs but not HDAC6 and HDAC10 (63). As was to be expected, their study did not show any effects of butyrate on the acetylation of α-tubulin (56). Further research is needed to understand butyrate-induced non-histone acetylation in mast cells and its biological consequences. Interestingly, the MAPK signaling pathway seems to react to changes in HDAC as phosphorylation of JNK, p38, and ERK1/2 was significantly inhibited in activated mast cells by both butyrate and TSA administration (56).

In summary, *in vitro* studies have suggested that both dietary fiber and its metabolites may regulate mast cell-associated diseases *via* their major inhibitory effects on mast cell activation and degranulation (Figure 1). Limited data provided by a few published studies have further suggested that some of these effects appear to be mediated *via* inhibition of the MAPK signaling pathway and reduced TF activity, which are known to modulate mast cell function. Further detailed investigations of the mechanisms by which butyrate inhibits mast cell activation are warranted as an important reference for interpreting the outcome of studies investigating the effects of dietary fiber and metabolites in mast cell-associated diseases.

## Dietary Fiber Interactions with Gastrointestinal Mast Cells

The GI is the primary organ to interact with dietary fiber. Although dietary fiber can, by definition, not be fermented before entering the large intestine, it exerts health benefits in the GI tract by regulating stool viscosity and increasing stool bulk. Patients with food allergies may also benefit from high-fiber concentrations in the small intestine, as pectin-rich fruits hamper allergen digestion by pepsin *in vivo* and *in vitro*, thus regulating allergic sensitization in atopic individuals (64). Most of the beneficial effects of dietary fiber on (GI) health, however, come from colon-derived microbial metabolites.

Throughout the GI tract, substantial quantities of mast cells are strategically positioned in the mucosa and submucosa (65). Therefore, their role as mediators of GI diseases, such as food allergy, has been well documented, and they have been proposed to contribute to the pathology of certain forms of colitis and Crohn’s disease (66–68). Indeed, intestinal mast cells can strongly regulate blood flow, smooth muscle contraction, GI barrier function, as well as the initiation of (allergic) inflammation (69). Moreover, mucosal type mast cell (a tryptase positive, chymase negative subtype) numbers may increase in certain types of (allergic) GI inflammation, whereas numbers of connective tissue type mast cells (a tryptase/chymase double positive mast cell subtype) are more stable (65, 70, 71). Due to their location in the gut, mast cells are under the influence of high concentrations of dietary fiber and its metabolites. Interestingly, the effects of high-fiber diets on these mast cell-associated diseases have been well documented and offer a promising future avenue for intervention (72, 73). Moreover, some direct effects of dietary fiber and metabolites on intestinal mast cell activation in these diseases have been reported as described below.

### Food Allergy

Reactions to food allergens are typically the result of intestinal mast cell activation and degranulation through food-specific IgE antibodies (30, 74). Symptoms related to food allergy can range in severity between mild (e.g., hives, abdominal pain, diarrhea, and wheezing) to life threatening (e.g., anaphylaxis). A recent study, in which more than 300,000 children participated, showed that the prevalence of food allergy in the US is 6.7%, with the most common food allergens being peanut (2.6%), milk (2.2%), egg (1.8%), shellfish (1.5%), and soy (0.7%) (75). On a global scale, the prevalence of food allergy is increasing, linked to industrialization and improved living conditions (76, 77). Strict allergen avoidance and oral immunotherapy remain common strategies to prevent food-dependent allergic reactions. Recently, the utilization of dietary fiber and its metabolites in the prevention or treatment of food allergies has received a lot of interest. Hogenkamp and colleagues (78) found impaired sensitization and attenuated allergic symptoms in the offspring of mice supplemented with dietary fiber, i.e., galacto-oligosaccharides (GOS), fructo-oligosaccharides (FOS), and pectin-derived acidic oligosaccharides. This suggests that dietary fiber can have significant effects on the development of food allergy in mice, even in the offspring of animals on high-fiber diets.

Several recent studies have provided insights into the mechanisms of action of dietary fiber and metabolites in allergic mice and humans. Tan et al. (79) reported that a high-fiber diet (enriched in guar gum and cellulose) protected mice against peanut allergy *via* altered gut microbiota and SCFA production. Anaphylaxis scores and IgE concentrations were decreased in mice fed with a high-fiber diet. However, whether this resulted in decreased mast cell activation was not reported. Interestingly, similar results were obtained by adding acetate or butyrate, but not propionate, to the drinking water for 3 weeks prior to allergen sensitization. These findings suggest that high-fiber diets can exert significant immunoregulatory effects though SCFA production. In a similar study by Kivit and colleagues (80), mice were fed prebiotic short-chain galacto- and FOS (scGOS/lcFOS, resembling the non-digestible oligosaccharides in human milk) and were orally sensitized to whey, a common food allergen. These mice displayed reduced acute hypersensitivity responses to the allergen, as measured by ear swelling, as well as serum mucosal mast cell protease-1 (mMCP-1) levels, both markers for mast cell degranulation.

However, in such studies diets are usually given for weeks before allergen sensitization. Their effects on allergic inflammation are, therefore, often attributed to beneficial immunoregulatory effects on sensitization and allergy development, rather than on the inflammatory response in established allergic diseases. Nonetheless, Kivit and colleagues (80) showed that scGOS/lcFOS diets upregulated galactin-9 levels in mice allergic to whey. Galactin-9 is a potent inhibitor of mast cell degranulation through blocking IgE–antigen complex formation (81), and high serum levels of galactin-9 are reportedly linked to a reduction of allergic inflammation and mast cell degranulation *in vivo* (80).

Ingestion of seaweeds commonly consumed in (Southeast) Asia are a rich source of polysaccharides (82), containing 33–50% more fiber than the higher plants commonly consumed in the West (Marine Algae Extracts: Processes, Products, and Applications, 2). Extracts of sulfated polysaccharides from seaweed have recently been shown to directly regulate food allergy symptoms *via* effects on mast cell activation *in vivo* and *in vitro*. Ngatu et al. (48) showed inhibition of β-hexosaminidase release in rat basophilic leukemia RBL-2H3 cells by sulfated polysaccharides. Xu et al. (49) extended these findings by reporting that *Eucheuma cottonii*-derived sulfated oligosaccharides (ESO) protected mice from food allergy and anaphylaxis symptoms in both a preventive manner as well as therapeutic manner when ESO was administered after initial food challenges. Serum concentrations of histamine, mMCP-1, and food allergen-specific IgE were all reduced in mice treated with a high-fiber diet. *In vitro* measurements showed that mast cell degranulation and cytokine production were inhibited by the sulfated oligosaccharides in a dose-dependent manner (49). These results complement earlier findings by the same group showing that sulfated polysaccharide from *Gracilaria lemaneiformis* had similar effects on food allergy symptoms in mice, as well as mast cell activation, although these effects were more modest (83). In their study, Castillo-Courtade and colleagues (50) tested the effects of two human milk oligosaccharides (HMOs) on immune responses in an ovalbumin-sensitized mouse model of food allergy. Both HMOs reduced diarrhea and hypothermia, as well as serum mMCP-1 concentrations in mice with food allergy. One of the HMOs, 6′-sialyllactose, was able to directly inhibit *in vitro* mast cell degranulation. Together, these studies suggest that dietary fiber, especially oligosaccharides and polysaccharides, may regulate food allergy symptoms in part directly *via* inhibition of mast cell activation—a potentially important finding for patients with food allergies. Food allergies are typically characterized by mast cell-induced intestinal mucosal permeability and inflammation of the ileum (84). Yet, the small intestine might miss out on much of the health benefits of high dietary metabolite concentrations, since they are primarily produced in the large intestine. Nonetheless, these studies show direct immunoregulatory effects of dietary fiber on mast cell function, thereby potentially influencing gut homeostasis in the small intestine as well.

Metabolites of dietary fiber have, justifiably, received significant attention in the search for possible explanations for the health benefits of dietary fiber. In addition to the indirect effects of dietary fiber (i.e., after fermentation in the large intestine, including upregulation of galactin-9 and SCFA production), fiber can have direct (i.e., prior to microbial fermentation) immunoregulatory effects on food allergy and mast cell activation. Thus, studies investigating the effects of dietary fiber on the onset and manifestation of allergies and other mast cell-associated diseases have to take into account that dietary fiber can directly influence immune responses, hereby regulating mast cell activation without intermediate factors.

### Inflammatory Bowel Disease

The inflammatory bowel diseases, ulcerative colitis (UC) and Crohn’s disease (CD), are characterized by chronic inflammation of the GI tract. Prevalence and incidence have stabilized in developed countries, but are increasing in newly industrialized countries (85), in a similar manner to atopic diseases. In UC, inflammation is initiated and maintained by an atypical Th2 response mediated by non-classical NKT cells. Typically, inflammation in UC occurs between the rectum and cecum, where the gut is exposed to high concentrations of metabolites of dietary fiber (86). CD on the other hand is thought to be triggered by an imbalance between Th1, Th17 cells, and Tregs, which initiates a mucosal inflammation that can occur throughout the GI tract (87). Although T cell subsets play a central role in the development of IBD, evidence indicating an important role of mast cells in the manifestation of IBD is accumulating (88, 89). In patients with UC or CD, histological and immuno-histochemical methods revealed mast cell degranulation in biopsies from inflamed tissue (90). Furthermore, mast cell numbers may be increased in fibrotic tissue of patients with CD (91), suggesting a functional role for mast cells in the pathogenesis of IBD. Indeed, mast cell expression of TNF, IL-16, and substance P, as well as histamine and tryptase levels were elevated in the mucosa of IBD patients (78). The contribution of mast cells to gut inflammation was further demonstrated in mast cell-deficient rats, which have significantly reduced microscopic mucosal damage in a DSS-induced colitis model (79). In wild type rats, mast cell counts correlated with local mucosal damage.

Nonetheless, direct activators of mast cells in IBD are not well defined, with currently no evidence indicating involvement of IgE-dependent mast cell activation. However, psychological stress, a known contributor to inflammation in IBD, caused significantly higher mast cell degranulation in patients with IBD (92). Indeed, corticotropin-releasing factor, a hormone known to be released in response to stress, increased intestinal paracellular permeability and gut inflammation *via* mast cell-dependent release of TNF and proteases (93). Finally, mast cells may exacerbate the symptoms of IBD following Ig-free L chain (Ig-fLC) activation. Ig-fLCs have been reported to bind to high affinity receptors on mast cells to induce degranulation upon second allergen encounter and an immediate allergic response (94). Interestingly, in a mast cell-dependent model for IBD, mice were sensitized with Ig-fLC followed by a rectal hapten challenge, after which they displayed mucosal mast cell activation as well as increased vascular permeability (95). Patients suffering from IBD show strongly increased Ig-fLC concentrations in serum, as well as in colon and ileum tissue, suggesting a possible role for mast cells in IBD *via* Ig-fLC sensitization.

Due to the location of the inflammation, novel therapies focus on using dietary fiber and metabolites to treat (96) or prevent (97) symptoms of IBD (72, 98, 99), including mast cell activation or recruitment. Van Hung et al. (100) recently showed that fermentable dietary fiber (guar gum) could reduce inflammation in colitic mice, possibly through the reported increase in fecal SCFA concentrations. High-fiber intake and metabolite production was inversely correlated with the loss of intestinal barrier function. Expression of pro-inflammatory cytokines, such as TNF, IL-6, and IL-17A, were downregulated due to the high-fiber diet, although the specific source of these cytokines was not mentioned. Similar studies investigating the capacity of dietary fiber to reduce inflammation in colitis models have reported that the beneficial effects of dietary fiber in UC are not dependent on species (101) or model (102), nor on the type of dietary fiber (91).

Although similar effects can be found among the different kinds of dietary fiber, their fermentability varies per type, and with it, the location in the gut in which they might exert their beneficial effects. Indeed, a combination of two dietary fibers with different fermentation patterns (FOS and resistant starch) led to synergistic prebiotic effects in colitis (103). These results can be translated into human studies, as supplementation with germinated barley foodstuff is able to reduce inflammation and improve the clinical activity index in UC patients (104, 105). Interestingly, fermentation of germinated barley foodstuff resulted in reduced colonic mast cell recruitment in colitic rats (106). The authors suggested that “*the most probable mechanism responsible for the amelioration of DSS-induced colitis is the generation of SCFAs derived from germinated barley foodstuff*.” Interestingly, administration of sodium butyrate to patients with distal UC indeed showed strong reduction of gut inflammation, although the role of mast cells in this context was not investigated (107). Since it is now known that mast cell activation can be inhibited by SCFAs *in vitro* as well as *in vivo* (55), the prediction that dietary fiber-derived SCFAs could regulate colitis *via* effects on mast cell activation seems plausible. Mast cells, dietary fiber, and its metabolites have thus established themselves as potential key modulators of inflammation in the large intestine, with the first signs of extensive interplay being reported (Table 2).

**Table 2:** Dietary fiber and metabolites in mast cell-associated diseases.

	Food allergy	Colitis/Crohn’s	Allergic asthma	Atopic dermatitis	*In vitro*	General health
Resistant oligosaccharides	↓ Allergic sensitization and symptoms in offspring (m) (78), ↓ acute hypersensitivity response (m) (80), ↓ diarrhea, hypothermia, and mucosal mast cell protease-1 (mMCP-1) (m) (50)		↓ Allergic airway inflammation (108, 109) and serum mMCP-1(m) (110)	↓ Incidence of allergic manifestations (111–113), ↓ clinical symptom score (114), ↓ plasma levels of total immunoglobulin E, IgG1, IgG2, IgG3 (115), and Ig-free L chain (116)	↓ Mouse mast cell degranulation directly (50, 108)	↑ SCFA production (7), ↑ galactin-9 expression (m) (80)

(Sulfated) Polysaccarides	↓ Food allergy and anaphylaxis symptoms (m) (49)		↓ Sneezing, nasal rubbing, and airway hyperresponsiveness(m) (117)		↓ Antigen-induced mouse mast cell degranulation (48, 83) and cytokine production (m) (49)	↑ SCFA production (7, 118)

Pectin			↓ Allergic inflammation (m) (119)			↓ Allergen digestion by pepsin (64)

(Guar) Gum	↓ Peanut allergy (m), altered microbiome, ↑ SCFA production (m) (79)	↓ Inflammation (m) (100)				↑ SCFA production (7, 100)

Resistant starch		↓ Inflammation (m), ↑ intestinal barrier function (m) (103)				↑ SCFA production (7)

Germinated barley foodstuff		↓ Clinical scores (104, 105), ↓ mast cell recruitment (r) (106)				↑ SCFA production (101)

Beta-glucan		↓ Mast cell-induced hyperpermeability of the ileum (120)				

Inulin			↓ Neutrophils, macrophages, lymphocytes, IL-8, and eNO in the sputum (121)			

Short-chain fatty acid (SCFA)	↓ Anaphylaxis (m) (79), ↓ mast cell activation and inflammatory mediator content (p) (55)	↓ Gut inflammation (107)	↓ Clinical symptoms (m) (122), ↑ hematopoiesis of common DC precursors and macrophage-DC precursors (m) (119)		↓ Mast cell degranulation and TNF-α production *via* Jun N-terminal kinase (52) or histone deacetylase (56)	

*m, in mouse; r, in rat; p, in pig*.

Studies regarding the effects of dietary fiber on colitis are relatively abundant, since microbial metabolites first become available at high concentrations in the proximal colon to exert potentially important effects on local health. Nonetheless, Mall et al. (120) recently reported that beta-glucan, a dietary fiber often found in oat and barley bran, strongly reduced mast cell-induced hyperpermeability of the ileum in patients with Crohn’s disease. In addition, they provided evidence that beta-glucan is partially able to inhibit *in vitro* mast cell activation, further strengthening the concept of a possible dietary fiber-metabolite-mast cell axis throughout the entire GI tract.

## Dietary Fiber in Mast Cell-Associated Airway Inflammation

Although limited in quantity, microbial metabolites remain key determinants in host–microbe mutualism outside of the intestinal tract, especially in well-perfused organs, such as the lungs. This was recently shown in a study by Halnes et al. (121), who reported that after ingestion of a meal high in soluble fiber (containing 3.5 g inulin and probiotics) the levels of neutrophils, macrophages, lymphocytes, IL-8, and eNO significantly decreased in the sputum of adults with stable asthma, as compared to the control group. Although a relatively small study, it reveals a potentially therapeutic effect of dietary fiber outside of the GI tract. This is in line with the general belief that a Mediterranean diet, typically high in fiber (due to fruits and vegetables) while low in saturated fats, can be a protective factor for wheezing and asthma (123, 124). Interestingly, patients with severe persistent asthma consume significantly less dietary fiber and more fat as compared to healthy controls (125).

The protective roll of dietary fiber in allergic airway diseases is further supported by mouse model studies, in which the effects of non-digestible oligosaccharides were tested on allergic airway inflammation and mast cell activation. Verheijden et al. reported that administration of GOS reduced house dust mite-driven allergic airway inflammation in mice, as measured by improved lung resistance, reduced cell numbers in bronchoalveolar lavage fluid (BALF), and lower cytokine production of lung homogenates (126). These effects could be explained by a strong inhibition of mast cell degranulation as assessed by measuring the mast cell degranulation marker mMCP-1 levels in serum (126). Sagar et al. (110) found similar effects in a more chronic model for airway inflammation. A diet rich in GOS, FOS, and pectin-derived acidic oligosaccharides (with *Bifidobacterium breve)* was able to reduce airway inflammation, as measured by BALF-cell count and cytokine production in mice challenged with ovalbumin (OVA). Remarkably, treatment with a high-fiber diet strongly reduced serum mMCP-1 levels in allergic mice as compared to control mice, while a similar reduction in mMCP-1 levels was detected in BALF of mice receiving the high-fiber diet. Direct effects of oligosaccharides on regulation of mast cell activation and degranulation in the airways have also been reported. Chung et al. (108) investigated the effects of chitosan oligosaccharides, an oligomer of β-(1–4)-linked d-glucosamine, on both allergic airway inflammation as well as on RBL-2H3 degranulation and cytokine production. Effects of low-molecular weight chitosan oligosaccharides on *in vivo* airway inflammation were consistent with the results of the previously mentioned studies, but were unique in reporting a reduction of IgE–antigen complex-stimulated RBL-2H3 degranulation, as well as diminished cytokine production (IL-4, IL-13, and TNF-α).

Fewer studies have focused on the effects of dietary fiber and its metabolites on mast cell-associated upper airway inflammation, such as allergic rhinitis (AR). Most studies only investigated possible correlations between Western (127, 128) and/or Mediterranean (129–131) diets and AR prevalence. Using a more mechanistic approach, Xie et al. (117) reported that rats orally administered dietary fiber in the form of *Cryptoporus* polysaccharides showed impaired symptoms of AR, including sneezing, nasal rubbing, and airway hyperresponsiveness. Wang et al. (122) directly administered sodium butyrate intranasally and found improved clinical symptoms in a mouse model of AR, most likely *via* HDAC inhibition.

Together, these studies implicate dietary fiber and metabolites as important regulators in the development and manifestation of allergic airway inflammation. Mast cells are essential facilitators of both upper and lower airway inflammation and have been shown to be under the direct influence of dietary fiber as well as its metabolites. Regulating mast cell activity *via* dietary fiber and metabolites may, therefore, offer novel therapeutic strategies in the treatment allergic asthma and rhinitis (Table 2).

In addition to the effects on mast cell-associated diseases already described, a more indirect pathway of dietary metabolites shaping immune homeostasis has emerged. Trompette et al. (119) found that mice on a high-fiber diet (supplemented with 30% cellulose or 30% pectin) showed significant differences in the ratio of Firmicutes to Bacteroidetes in their gut and lung microbiome. These mice also had increased levels of circulating SCFAs and were protected against allergic inflammation in the lung. Similar results were obtained in mice treated with the SCFA propionate (119). Remarkably, these effects were not associated with increased FoxP3 + CD25+ regulatory CD4+ T cells. In propionate-treated mice, recruitment of dendritic cells (DCs) and CD4+ T cells to the lung-draining lymph nodes was unaffected after 1–4 days. The activation state of these cells was similarly unaffected, with exceptions during the later stage of inflammation (day 4), suggesting that in this model the beneficial effects of propionate on airway inflammation are exerted by different means and/or cell types.

Elevated SCFAs concentrations were found in the circulation of high-fiber fed mice, but were undetectable in the lung, which could, at least in part, explain a lack of effects in the lung-draining lymph nodes themselves (119). Rather, SCFAs might influence hematopoiesis in the bloodstream and/or bone marrow. Trompette and colleagues reported that propionate treatment enhanced hematopoiesis of common DC precursors and macrophage-DC precursors, but did not mention any changes in hematopoietic differentiation of other cell types (119). Taken together, current evidence suggests that dietary fiber metabolites may influence allergic airway inflammation *via* altered immune cell differentiation in bone marrow and/or blood circulation. Similar to DCs and macrophages, mast cell precursors originate from multipotent hematopoietic progenitor cells in the bone marrow, after which they enter the blood stream to mature in (mucosal) tissues (132, 133). This indicates that the effects of SCFAs on DCs and macrophages may translate to mast cell development and recruitment as well. Interestingly, it was recently reported that the administration of *Bifidobacterium breve*, a SCFA-producing bacteria strain (134), was able to regulate mast cell migration and activation in a mouse model of chronic allergic asthma (135). Interestingly, mast cells cultured from the bone marrow of mice receiving this SCFA-producing bacterial strain, displayed impaired IgE-mediated degranulation, which was not caused by reduced mast cell maturation (135). These data may indicate that microbial metabolites can influence mast cell migration and function *via* hematopoiesis in the bone marrow.

More research regarding the effects of dietary fiber and metabolites on mast cell activation in asthma is needed, including studying the possible effects of SCFAs on mast cell progenitors within this context.

## Atopic Dermatitis (AD)

Affecting up to 20% of children and up to 3% of adults in certain countries (136), AD is responsible for a significant social, health, and financial burden. Although a role for mast cells in AD has been established (137, 138), reports on the effects of dietary fiber and metabolites on skin inflammation have been divergent and sometimes even contradictory. Being located distant from the large intestine, the skin ordinarily contains neither high concentrations of dietary fiber nor its metabolites, making it an unlikely organ to be affected by gut microbial products. Nonetheless, there is emerging evidence suggesting that high-fiber diets can help to prevent and treat AD in mice and young infants, although the mechanism of this effect requires further investigation. AD is typically diagnosed in (very) young infants and considered the first manifestation of the atopic march (139). Studies investigating the effects of dietary fiber on AD, therefore, mainly use scGOS/lcFOS resembling non-digestible oligosaccharides in human milk, often the first type of diet young infants receive.

Arslanoglu and colleagues (111) studied the protective effects of scGOS/lcFOS supplementation in young infants with parental history of atopy and assessed the incidence of allergic manifestations during 2 years. Within this period, children receiving the scGOS/lcFOS supplementation had significantly lower incidence of allergic manifestations, including AD. They also reported a decline in recurrent wheezing and allergic urticaria, in addition to fewer respiratory tract infections, fever episodes, and antibiotic prescriptions. Interestingly, most of these beneficial health effects persisted until the age of five, as reported in their follow-up study (112). Grüber et al. showed a similar decrease in AD prevalence in low-atopy-risk children, in a somewhat larger study (>300) (113). Using only fructo-oligosaccharide for the treatment of AD was also shown to be effective (114). The AD symptom score (SCORAD) was significantly lower following administration of Kestose, a fructo-oligosaccharide, for 12 weeks. An interesting study by van Hoffen et al. showed that GOS/FOS supplementation in young infants could lead to a significant reduction in plasma levels of total IgE, IgG1, IgG2, and IgG3, but not IgG4 (115). These data suggest that GOS/FOS supplementation induces a beneficial antibody profile, potentially modulating the outcome of allergic manifestations later in life.

Although concentrations of Ig-fLC are often not measured, they are significantly associated with allergic and non-atopic rhinitis, asthma, food allergy, and colitis (95, 140, 141). In line with previous reports, Schouten et al. found that plasma levels of Ig-fLC were elevated in patients with AD, but for the first time showed attenuated Ig-fLC production in young infants at risk receiving a scGOS/lcFOS mixture (116). This represents a potentially important finding for patients suffering from mast cell-associated diseases, since Ig-fLCs have been shown to induce mast cell activation (as described above) (94).

In summary, these studies suggest that dietary fiber may influence the development and manifestation of AD in patients, although further research into the precise role of mast cells in this context is required. Last, it should not go without mentioning that intervention diets based on oligosaccharide supplementation, administered to young infants, do not always succeed in either treatment (142) or prevention (143, 144) of AD.

## Concluding Remarks and Future Perspectives

Mast cells have been shown to play a crucial role in the manifestation of allergic and non-allergic diseases. Many of these mast cell-associated diseases exhibit a significant increase in prevalence on a global scale, closely following the development of industrialization and improved living conditions. Dietary fiber and its metabolites are thought to play an important role in the development and manifestation of these diseases, as recent research has provided evidence of significant health benefits of dietary fiber and its metabolites in the prevention and treatment of mast cell-associated diseases.

Here, we have summarized the known effects of these dietary fibers and metabolites on mast cell activation *in vitro* and *in vivo* (Table 2). Consistent with the reported health benefits on other immune cells (145), dietary fiber (especially polysaccharides and oligosaccharides) and metabolites (SCFAs) can regulate mast cell function. Mast cell activation can be downregulated by pretreatment with these substances, *via* inhibition of various components of the MAPK signaling pathway and a subsequent reduction in transcription factor activity. Furthermore, both butyrate and the activation of the MAPK signaling pathway can induce chromatin modifications *via* the regulation of HDAC activity and its intracellular localization. In addition, diets high in fiber can prevent sensitization of mast cells either by inhibiting allergen digestion or by upregulating galactin-9 expression in mice, thus blocking the formation of IgE–antigen complexes.

Although these results are promising and may provide new insights regarding novel approaches to target mast cell function, certain key mechanisms mediating the inhibitory effects on mast cells remain unclear. Inhibition of late-phase mast cell activation by dietary fiber and butyrate, characterized by reduced mediator synthesis and secretion, can be explained by their effects on MAPK phosphorylation and transcription factor activity. However, modulation of these pathways by dietary components cannot account for all of the inhibitory effects observed with mast cell degranulation. The cascade leading up to mast cell degranulation is a complex process upstream of MAPK signaling, mainly involving PLCγ and PKC activation followed by an increase in calcium mobilization. Indeed, mast cell degranulation is not known to be affected by inhibition of the MAPK signaling pathway (39). Possible alternative mechanisms could involve modifications of the mast cell proteome and epigenome by dietary components (e.g., by promoting acetylation). Since butyrate is a potent HDAC inhibitor, it could impact many key gene regulatory processes modulating mast cell activity through histone acetylation, including transcription factor binding and transcription activation. Potential therapeutic implications of butyrate-induced epigenome modifications have been described (141), but no direct link of such therapeutic effects with regulation of mast cell function has been established. The *de novo* synthesis of new proteins may not be required, as the functions of the expressed proteins in the cell could be readily modified by butyrate-regulated HDACs and post-translational acetylation is known to regulate protein function (61). However, the effects of non-histone protein acetylation on mast cell function remain unclear.

Finally, dietary fiber and its metabolites may exert their effects on mast cells in an indirect manner. Very recently, the function of other immune cells, such as group 2 innate lymphoid cells (ILC2s), have shown to be subject to the effects of butyrate (146). Cell activation, local cytokine production, and secretion as well as cell to cell contact with/by other immune cells can modulate mast cell responses (147). Therefore, butyrate may regulate mast cell function indirectly *via* secondary pathways as well, e.g., *via* IL-9-producing ILC2s (148). Taken together, mast cell function has been shown to be susceptible to the immunoregulatory effects of dietary fiber and butyrate. Further understanding of the mechanisms by which dietary fiber and butyrate can regulate mast cell activation may help in the future development of novel approaches for the prevention and treatment of mast cell-associated diseases.

## Author Contributions

JF performed the literature research and writing of the manuscript. RS, FR, S-YT, RH, SG, and MM edited and shaped the manuscript. All authors read and approved the final manuscript.

## Conflict of Interest Statement

The authors declare that the research was conducted in the absence of any commercial or financial relationships that could be construed as a potential conflict of interest. The handling Editor declared a past co-authorship with one of the authors [FR].

## References

[1] Maslowski KM Mackay CR. Diet, gut microbiota and immune responses. Nat Immunol (2011) 12:5–9. 10.1038/ni0111-5 21169997

[2] Thorburn AN Macia L Mackay CR Diet, metabolites, and “western-lifestyle” inflammatory diseases. Immunity (2014) 40:833–42. 10.1016/j.immuni.2014.05.014 24950203

[3] McKenzie C Tan J Macia L Mackay CR. The nutrition-gut microbiome-physiology axis and allergic diseases. Immunol Rev (2017) 278:277–95. 10.1111/imr.12556 28658542

[4] Devereux G. The increase in the prevalence of asthma and allergy: food for thought. Nat Rev Immunol (2006) 6:869–74. 10.1038/nri1958 17063187

[5] Cochrane S Beyer K Clausen M Wjst M Hiller R Nicoletti C Factors influencing the incidence and prevalence of food allergy. Allergy (2009) 64:1246–55. 10.1111/j.1398-9995.2009.02128.x 19663867

[6] Mutius von E Weiland SK Fritzsch C Duhme H Keil U. Increasing prevalence of hay fever and atopy among children in Leipzig, East Germany. Lancet (1998) 351:862–6. 10.1016/S0140-6736(97)10100-3 9525363

[7] Tan J McKenzie C Potamitis M Thorburn AN Mackay CR Macia L The role of short-chain fatty acids in health and disease. Adv Immunol (2014) 121:91–119. 10.1016/B978-0-12-800100-4.00003-9 24388214

[8] Høverstad T Midtvedt T. Short-chain fatty acids in germfree mice and rats. J Nutr (1986) 116:1772–6. 10.1093/jn/116.9.1772 3761032

[9] Ley RE Turnbaugh PJ Klein S Gordon JI. Microbial ecology: human gut microbes associated with obesity. Nature (2006) 444:1022–3. 10.1038/4441022a 17183309

[10] Vitaglione P Mennella I Ferracane R Rivellese AA Giacco R Ercolini D Whole-grain wheat consumption reduces inflammation in a randomized controlled trial on overweight and obese subjects with unhealthy dietary and lifestyle behaviors: role of polyphenols bound to cereal dietary fiber. Am J Clin Nutr (2015) 101:251–61. 10.3945/ajcn.114.088120 25646321

[11] Davis HC. Can the gastrointestinal microbiota be modulated by dietary fibre to treat obesity? Ir J Med Sci (2018) 187(2):393–402. 10.1007/s11845-017-1686-9 29038988

[12] Melli LCFL do Carmo-Rodrigues MS Araújo-Filho HB Solé D de Morais MB Intestinal microbiota and allergic diseases: a systematic review. Allergol Immunopathol (Madr) (2016) 44:177–88. 10.1016/j.aller.2015.01.013 25985709

[13] Lynch SV. Gut microbiota and allergic disease. New insights. Ann Am Thorac Soc (2016) 13(Suppl 1):S51–4. 10.1513/AnnalsATS.201507-451MG 27027953 PMC5015732

[14] Kyburz A Müller A. The gastrointestinal tract microbiota and allergic diseases. Dig Dis (2016) 34:230–43. 10.1159/000443357 27028536

[15] AACC Report. The definition of dietary fiber. Cereal Foods World (2001) 46:3.

[16] Lattimer JM Haub MD. Effects of dietary fiber and its components on metabolic health. Nutrients (2010) 2:1266–89. 10.3390/nu2121266 22254008 PMC3257631

[17] Shibata N Kunisawa J Kiyono H. Dietary and microbial metabolites in the regulation of host immunity. Front Microbiol (2017) 8:2171. 10.3389/fmicb.2017.02171 29163449 PMC5681998

[18] El-Zaatari M Kao JY. Role of dietary metabolites in regulating the host immune response in gastrointestinal disease. Front Immunol (2017) 8:51. 10.3389/fimmu.2017.00051 28191010 PMC5269446

[19] Le Poul E Loison C Struyf S Springael J-Y Lannoy V Decobecq M-E Functional characterization of human receptors for short chain fatty acids and their role in polymorphonuclear cell activation. J Biol Chem (2003) 278:25481–9. 10.1074/jbc.M301403200 12711604

[20] Maslowski KM Vieira AT Ng A Kranich J Sierro F Yu D Regulation of inflammatory responses by gut microbiota and chemoattractant receptor GPR43. Nature (2009) 461:1282–6. 10.1038/nature08530 19865172 PMC3256734

[21] Sina C Gavrilova O Förster M Till A Derer S Hildebrand F G protein-coupled receptor 43 is essential for neutrophil recruitment during intestinal inflammation. J Immunol (2009) 183:7514–22. 10.4049/jimmunol.0900063 19917676

[22] Alex S Lange K Amolo T Grinstead JS Haakonsson AK Szalowska E Short-chain fatty acids stimulate angiopoietin-like 4 synthesis in human colon adenocarcinoma cells by activating peroxisome proliferator-activated receptor γ. Mol Cell Biol (2013) 33:1303–16. 10.1128/MCB.00858-12 23339868 PMC3624264

[23] Daynes RA Jones DC. Emerging roles of PPARS in inflammation and immunity. Nat Rev Immunol (2002) 2:748–59. 10.1038/nri912 12360213

[24] Aoyama M Kotani J Usami M. Butyrate and propionate induced activated or non-activated neutrophil apoptosis via HDAC inhibitor activity but without activating GPR-41/GPR-43 pathways. Nutrition (2010) 26:653–61. 10.1016/j.nut.2009.07.006 20004081

[25] Jansen MS Nagel SC Miranda PJ Lobenhofer EK Afshari CA McDonnell DP. Short-chain fatty acids enhance nuclear receptor activity through mitogen-activated protein kinase activation and histone deacetylase inhibition. Proc Natl Acad Sci U S A (2004) 101:7199–204. 10.1073/pnas.0402014101 15103026 PMC406489

[26] Kim HJ Rowe M Ren M Hong JS Chen PS Chuang DM. Histone deacetylase inhibitors exhibit anti-inflammatory and neuroprotective effects in a rat permanent ischemic model of stroke: multiple mechanisms of action. J Pharmacol Exp Ther (2007) 321:892–901. 10.1124/jpet.107.120188 17371805

[27] Hasselgren P-O Ubiquitination, phosphorylation, and acetylation—triple threat in muscle wasting. J Cell Physiol (2007) 213:679–89. 10.1002/jcp.21190 17657723

[28] Cummings JH Hill MJ Bone ES Branch WJ Jenkins DJA The effect of meat protein and dietary fiber on colonic function and metabolism II. Bacterial metabolites in feces and urine. Am J Clin Nutr (1979) 32:2094–101. 10.1093/ajcn/32.10.2094 484528

[29] Cummings JH Pomare EW Branch WJ Naylor CP Macfarlane GT. Short chain fatty acids in human large intestine, portal, hepatic and venous blood. Gut (1987) 28:1221–7. 10.1136/gut.28.10.1221 3678950 PMC1433442

[30] Galli SJ Tsai M. IgE and mast cells in allergic disease. Nat Med (2012) 18:693–704. 10.1038/nm.2755 22561833 PMC3597223

[31] Krystel-Whittemore M Dileepan KN Wood JG. Mast cell: a multi-functional master cell. Front Immunol (2015) 6:620. 10.3389/fimmu.2015.00620 26779180 PMC4701915

[32] Siebenhaar F Redegeld FA Bischoff SC Gibbs BF Maurer M. Mast cells as drivers of disease and therapeutic targets. Trends Immunol (2018) 39:151–62. 10.1016/j.it.2017.10.005 29196147

[33] Furuta GT Schmidt-Choudhury A Wang MY Wang ZS Lu L Furlano RI Mast cell-dependent tumor necrosis factor alpha production participates in allergic gastric inflammation in mice. Gastroenterology (1997) 113:1560–9. 10.1053/gast.1997.v113.pm9352858 9352858

[34] Bischoff SC Lorentz A Schwengberg S Weier G Raab R Manns MP. Mast cells are an important cellular source of tumour necrosis factor alpha in human intestinal tissue. Gut (1999) 44:643–52. 10.1136/gut.44.5.643 10205200 PMC1727516

[35] Krüger-Krasagakes S Möller A Kolde G Lippert U Weber M Henz BM. Production of interleukin-6 by human mast cells and basophilic cells. J Invest Dermatol (1996) 106:75–9. 10.1111/1523-1747.ep12327815 8592085

[36] Gilfillan AM Tkaczyk C. Integrated signalling pathways for mast-cell activation. Nat Rev Immunol (2006) 6:218–30. 10.1038/nri1782 16470226

[37] Plotnikov A Zehorai E Procaccia S Seger R. The MAPK cascades: signaling components, nuclear roles and mechanisms of nuclear translocation. Biochim Biophys Acta (2011) 1813:1619–33. 10.1016/j.bbamcr.2010.12.012 21167873

[38] Azzolina A Guarneri P Lampiasi N. Involvement of p38 and JNK MAPKs pathways in substance P-induced production of TNF-alpha by peritoneal mast cells. Cytokine (2002) 18:72–80. 10.1006/cyto.2002.0879 12096921

[39] Koranteng RD Swindle EJ Davis BJ Dearman RJ Kimber I Flanagan BF Differential regulation of mast cell cytokines by both dexamethasone and the p38 mitogen-activated protein kinase (MAPK) inhibitor SB203580. Clin Exp Immunol (2004) 137:81–7. 10.1111/j.1365-2249.2004.02510.x 15196247 PMC1809098

[40] Cildir G Pant H Lopez AF Tergaonkar V. The transcriptional program, functional heterogeneity, and clinical targeting of mast cells. J Exp Med (2017) 214:2491–506. 10.1084/jem.20170910 28811324 PMC5584128

[41] Gaudenzio N Sibilano R Marichal T Starkl P Reber LL Cenac N Different activation signals induce distinct mast cell degranulation strategies. Different activation signals induce distinct mast cell degranulation strategies. J Clin Invest (2016) 126:3981–98. 10.1172/JCI85538 27643442 PMC5096814

[42] Ali H. Regulation of human mast cell and basophil function by anaphylatoxins C3a and C5a. Immunol Lett (2010) 128:36–45. 10.1016/j.imlet.2009.10.007 19895849 PMC2815128

[43] Heimbach L Li Z Berkowitz P Zhao M Li N Rubenstein DS The C5a receptor on mast cells is critical for the autoimmune skin-blistering disease bullous pemphigoid. J Biol Chem (2011) 286:15003–9. 10.1074/jbc.M111.221036 21393236 PMC3083169

[44] Macian F. NFAT proteins: key regulators of T-cell development and function. Nat Rev Immunol (2005) 5:472–84. 10.1038/nri1632 15928679

[45] Cyert MS Regulation of nuclear localization during signaling. J Biol Chem (2001) 276:20805–8. 10.1074/jbc.R100012200 11303030

[46] Zhou X Richon VM Wang AH Yang XJ Rifkind RA Marks PA. Histone deacetylase 4 associates with extracellular signal-regulated kinases 1 and 2, and its cellular localization is regulated by oncogenic Ras. Proc Natl Acad Sci U S A (2000) 97:14329–33. 10.1073/pnas.250494697 11114188 PMC18918

[47] Mao W-A Sun Y-Y Mao J-Y Wang L Zhang J Zhou J Inhibitory effects of angelica polysaccharide on activation of mast cells. Evid Based Complement Alternat Med (2016) 2016:1–10. 10.1155/2016/6063475 27200102 PMC4854997

[48] Ngatu NR Tanaka M Ikeda M Inoue M Kanbara S Nojima S. Sujiaonori-derived algal biomaterials inhibit allergic reaction in allergen-sensitized RBL-2H3 cell line and improve skin health in humans. J Funct Biomater (2017) 8:37. 10.3390/jfb8030037 28850069 PMC5618288

[49] Xu S-S Liu Q-M Xiao A-F Maleki SJ Alcocer M Gao Y-Y *Eucheuma cottonii* sulfated oligosaccharides decrease food allergic responses in animal models by up-regulating regulatory T (Treg) cells. J Agric Food Chem (2017) 65:3212–22. 10.1021/acs.jafc.7b00389 28359154

[50] Castillo-Courtade L Han S Lee S Mian FM Buck R Forsythe P. Attenuation of food allergy symptoms following treatment with human milk oligosaccharides in a mouse model. Allergy (2015) 70:1091–102. 10.1111/all.12650 25966668

[51] Galli SJ Dvorak AM Marcum JA Ishizaka T Nabel G Der Simonian H Mast cell clones: a model for the analysis of cellular maturation. J Cell Biol (1982) 95:435–44. 10.1083/jcb.95.2.435 6216259 PMC2112969

[52] Diakos C Prieschl EE Säemann MD Böhmig GA Csonga R Sobanov Y n-Butyrate inhibits Jun NH(2)-terminal kinase activation and cytokine transcription in mast cells. Biochem Biophys Res Commun (2006) 349:863–8. 10.1016/j.bbrc.2006.08.117 16949031

[53] Baumruker T Pendl GG Prieschi EE Gene regulation after Fc epsilon Rl stimulation in the murine mast cell line CPII. Int Arch Allergy Immunol (2004) 113:39–41. 10.1159/000237502 9130478

[54] Baumruker T Csonga R Jaksche D Novotny V Prieschl EE TNF-alpha and IL-5 gene induction in IgE plus antigen-stimulated mast cells require common and distinct signaling pathways. Int Arch Allergy Immunol (1999) 118:108–11. 10.1159/000024042 10224353

[55] Wang CC Wu H Lin FH Gong R Xie F Peng Y Sodium butyrate enhances intestinal integrity, inhibits mast cell activation, inflammatory mediator production and JNK signaling pathway in weaned pigs. Innate Immun (2017) 24:40–6. 10.1177/1753425917741970 29183244 PMC6830759

[56] Zhang H Du M Yang Q Zhu M-J. Butyrate suppresses murine mast cell proliferation and cytokine production through inhibiting histone deacetylase. J Nutr Biochem (2016) 27:299–306. 10.1016/j.jnutbio.2015.09.020 26601598

[57] Fann M Godlove JM Catalfamo M Wood WH Chrest FJ Chun N Histone acetylation is associated with differential gene expression in the rapid and robust memory CD8(+) T-cell response. Blood (2006) 108:3363–70. 10.1182/blood-2006-02-005520 16868257 PMC1895425

[58] Ghare SS Joshi-Barve S Moghe A Patil M Barker DF Gobejishvili L Coordinated histone H3 methylation and acetylation regulate physiologic and pathologic fas ligand gene expression in human CD4+ T cells. J Immunol (2014) 193:412–21. 10.4049/jimmunol.1400055 24899502 PMC5096587

[59] Johnson ES Kornbluth S. Life, death, and the metabolically controlled protein acetylome. Curr Opin Cell Biol (2012) 24:876–80. 10.1016/j.ceb.2012.10.002 23103123

[60] Choudhary C Kumar C Gnad F Nielsen ML Rehman M Walther TC Lysine acetylation targets protein complexes and co-regulates major cellular functions. Science (2009) 325:834–40. 10.1126/science.1175371 19608861

[61] Kim E Bisson WH Löhr CV Williams DE Ho E Dashwood RH Histone and non-histone targets of dietary deacetylase inhibitors. Curr Top Med Chem (2016) 16:714–31. 10.2174/1568026615666150825125857 26303421 PMC5087604

[62] Hubbert C Guardiola A Shao R Kawaguchi Y Ito A Nixon A HDAC6 is a microtubule-associated deacetylase. Nature (2002) 417:455–8. 10.1038/417455a 12024216

[63] Davie JR Inhibition of histone deacetylase activity by butyrate. J Nutrition (2003) 133:2485S–93S. 10.1093/jn/133.7.2485S 12840228

[64] Polovic N Blanusa M Gavrovic-Jankulovic M Atanaskovic-Markovic M Burazer L Jankov R A matrix effect in pectin-rich fruits hampers digestion of allergen by pepsin in vivo and in vitro. Clin Exp Allergy (2007) 37:764–71. 10.1111/j.1365-2222.2007.02703.x 17456224

[65] Jakate S Demeo M John R Tobin M Keshavarzian A. Mastocytic enterocolitis: increased mucosal mast cells in chronic intractable diarrhea. Arch Pathol Lab Med (2006) 130:362–7. 10.1043/1543-2165(2006)130[362:MEIMMC]2.0.CO;2 16519565

[66] Ramsay DB Stephen S Borum M Voltaggio L Doman DB. Mast cells in gastrointestinal disease. Gastroenterol Hepatol (N Y) (2010) 6:772–7. 21301631 PMC3033552

[67] Kraneveld AD Sagar S Garssen J Folkerts G. The two faces of mast cells in food allergy and allergic asthma: the possible concept of Yin Yang. Biochim Biophys Acta (2012) 1822:93–9. 10.1016/j.bbadis.2011.06.013 21757003

[68] Bischoff SC. Mast cells in gastrointestinal disorders. Eur J Pharmacol (2016) 778:139–45. 10.1016/j.ejphar.2016.02.018 26852959

[69] Bischoff SC. Physiological and pathophysiological functions of intestinal mast cells. Semin Immunopathol (2009) 31:185–205. 10.1007/s00281-009-0165-4 19533134

[70] Guilarte M Santos J de Torres I Alonso C Vicario M Ramos L Diarrhoea-predominant IBS patients show mast cell activation and hyperplasia in the jejunum. Gut (2007) 56:203–9. 10.1136/gut.2006.100594 17005763 PMC1856785

[71] Weston AP Biddle WL Bhatia PS Miner PB Jr. Terminal ileal mucosal mast cells in irritable bowel syndrome. Dig Dis Sci (1993) 38:1590–5. 10.1007/BF01303164 8359068

[72] Viladomiu M Hontecillas R Yuan L Lu P Bassaganya-Riera J. Nutritional protective mechanisms against gut inflammation. J Nutr Biochem (2013) 24:929–39. 10.1016/j.jnutbio.2013.01.006 23541470 PMC3730123

[73] Gentschew L Ferguson LR. Role of nutrition and microbiota in susceptibility to inflammatory bowel diseases. Mol Nutr Food Res (2012) 56:524–35. 10.1002/mnfr.201100630 22495981

[74] Finkelman FD Anaphylaxis: lessons from mouse models. J Allergy ClinImmunol (2007) 120:506–15. 10.1016/j.jaci.2007.07.033 17765751

[75] Hill DA Grundmeier RW Ram G Spergel JM. The epidemiologic characteristics of healthcare provider-diagnosed eczema, asthma, allergic rhinitis, and food allergy in children: a retrospective cohort study. BMC Pediatr (2016) 16:133. 10.1186/s12887-016-0673-z 27542726 PMC4992234

[76] Fisher RJ Characterization of rat liver mitochondria depleted of inner membrane components by chloramphenicol treatment of regenerating rat liver. Arch Biochem Biophys (1976) 172:611–7. 10.1016/0003-9861(76)90115-6 130832

[77] Yu W Freeland DMH Nadeau KC. Food allergy: immune mechanisms, diagnosis and immunotherapy. Nat Rev Immunol (2016) 16:751–65. 10.1038/nri.2016.111 27795547 PMC5123910

[78] Hogenkamp A Knippels LM Garssen J van Esch BC. Supplementation of mice with specific nondigestible oligosaccharides during pregnancy or lactation leads to diminished sensitization and allergy in the female offspring. J Nutr (2015) 145:996–1002. 10.3945/jn.115.210401 25833889

[79] Tan J McKenzie C Vuillermin PJ Goverse G Vinuesa CG Mebius RE Dietary fiber and bacterial SCFA enhance oral tolerance and protect against food allergy through diverse cellular pathways. Cell Rep (2016) 15:2809–24. 10.1016/j.celrep.2016.05.047 27332875

[80] Kivit S Saeland E Kraneveld AD Kant HJG Schouten B Esch BCAM Galectin-9 induced by dietary synbiotics is involved in suppression of allergic symptoms in mice and humans. Allergy (2012) 67:343–52. 10.1111/j.1398-9995.2011.02771.x 22229637

[81] Niki T Tsutsui S Hirose S Aradono S Sugimoto Y Takeshita K Galectin-9 is a high affinity IgE-binding lectin with anti-allergic effect by blocking IgE-antigen complex formation. J Biol Chem (2009) 284:32344–52. 10.1074/jbc.M109.035196 19776007 PMC2781649

[82] Ngo D-H Kim S-K. Sulfated polysaccharides as bioactive agents from marine algae. Int J Biol Macromol (2013) 62:70–5. 10.1016/j.ijbiomac.2013.08.036 23994790

[83] Liu Q-M Yang Y Maleki SJ Alcocer M Xu S-S Shi C-L Anti-food allergic activity of sulfated polysaccharide from *Gracilaria lemaneiformisis* dependent on immunosuppression and inhibition of p38 MAPK. J Agric Food Chem (2016) 64:4536–44. 10.1021/acs.jafc.6b01086 27186807

[84] Russell DA Mast cells in the regulation of intestinal electrolyte transport. Am J Physiol (1986) 251:G253–62. 10.1152/ajpgi.1986.251.2.G253 2426973

[85] Ng SC Shi HY Hamidi N Underwood FE Tang W Benchimol EI Worldwide incidence and prevalence of inflammatory bowel disease in the 21st century: a systematic review of population-based studies. Lancet (2018) 390:2769–78. 10.1016/S0140-6736(17)32448-0 29050646

[86] Ungaro R Mehandru S Allen PB Peyrin-Biroulet L Colombel J-F. Ulcerative colitis. Lancet (2017) 389:1756–70. 10.1016/S0140-6736(16)32126-2 27914657 PMC6487890

[87] Baumgart DC Sandborn WJ. Crohn’s disease. Lancet (2012) 380:1590–605. 10.1016/S0140-6736(12)60026-9 22914295

[88] Hamilton MJ Frei SM Stevens RL. The multifaceted mast cell in inflammatory bowel disease. Inflamm Bowel Dis (2014) 20:2364–78. 10.1097/MIB.0000000000000142 25401721 PMC4428674

[89] Boeckxstaens G Mast cells and inflammatory bowel disease. Curr Opin Pharmacol (2015) 25:45–9. 10.1016/j.coph.2015.11.005 26629596

[90] Bischoff SC Wedemeyer J Herrmann A Meier PN Trautwein C Cetin Y Quantitative assessment of intestinal eosinophils and mast cells in inflammatory bowel disease. Histopathology (1996) 28:1–13. 10.1046/j.1365-2559.1996.262309.x 8838115

[91] Gelbmann CM Mestermann S Gross V Kollinger M Scholmerich J Falk W. Strictures in Crohn’s disease are characterised by an accumulation of mast cells colocalised with laminin but not with fibronectin or vitronectin. Gut (1999) 45:210–7. 10.1136/gut.45.2.210 10403732 PMC1727598

[92] Farhadi A Keshavarzian A Fields JZ Jakate S Shaikh M Banan A. Reduced immunostaining for c-kit receptors in mucosal mast cells in inflammatory bowel disease. J Gastroenterol Hepatol (2007) 22:2338–43. 10.1111/j.1440-1746.2007.05011.x 17645464

[93] Overman EL Rivier JE Moeser AJ. CRF induces intestinal epithelial barrier injury via the release of mast cell proteases and TNF-α. PLoS One (2012) 7:e39935. 10.1371/journal.pone.0039935 22768175 PMC3386952

[94] Redegeld FA van der Heijden MW Kool M Heijdra BM Garssen J Kraneveld AD Immunoglobulin-free light chains elicit immediate hypersensitivity-like responses. Nat Med (2002) 8:694–701. 10.1038/nm722 12068287

[95] Rijnierse A Redegeld FA Blokhuis BR Van der Heijden MW Velde Te AA Pronk I Ig-free light chains play a crucial role in murine mast cell-dependent colitis and are associated with human inflammatory bowel diseases. J Immunol (2010) 185:653–9. 10.4049/jimmunol.0901129 20505143

[96] Cabré E Domènech E. Impact of environmental and dietary factors on the course of inflammatory bowel disease. World J Gastroenterol (2012) 18:3814–22. 10.3748/wjg.v18.i29.3814 22876032 PMC3413052

[97] Liu X Wu Y Li F Zhang D. Dietary fiber intake reduces risk of inflammatory bowel disease: result from a meta-analysis. Nutr Res (2015) 35:753–8. 10.1016/j.nutres.2015.05.021 26126709

[98] Owczarek D Rodacki T Domagała-Rodacka R Cibor D Mach T. Diet and nutritional factors in inflammatory bowel diseases. World J Gastroenterol (2016) 22:895–905. 10.3748/wjg.v22.i3.895 26811635 PMC4716043

[99] Charlebois A Rosenfeld G Bressler B. The impact of dietary interventions on the symptoms of inflammatory bowel disease: a systematic review. Crit Rev Food Sci Nutr (2016) 56:1370–8. 10.1080/10408398.2012.760515 25569442

[100] Hung TV Suzuki T. Dietary fermentable fiber reduces intestinal barrier defects and inflammation in colitic mice. J Nutr (2016) 146:1970–9. 10.3945/jn.116.232538 27605405

[101] Araki Y Fujiyama Y Andoh A Koyama S Kanauchi O Bamba T. The dietary combination of germinated barley foodstuff plus *Clostridium butyricum* suppresses the dextran sulfate sodium-induced experimental colitis in rats. Scand J Gastroenterol (2009) 35:1060–7. 10.1080/003655200451180 11099059

[102] Jiminez JA Uwiera TC Abbott DW Uwiera RRE Inglis GD. Impacts of resistant starch and wheat bran consumption on enteric inflammation in relation to colonic bacterial community structures and short-chain fatty acid concentrations in mice. Gut Pathog (2016) 8:576. 10.1186/s13099-016-0149-6 28031748 PMC5178079

[103] Rodríguez-Cabezas ME Camuesco D Arribas B Garrido-Mesa N Comalada M Bailón E The combination of fructooligosaccharides and resistant starch shows prebiotic additive effects in rats. Clin Nutr (2010) 29:832–9. 10.1016/j.clnu.2010.05.005 20605664

[104] Kanauchi O Mitsuyama K Homma T Takahama K Fujiyama Y Andoh A Treatment of ulcerative colitis patients by long-term administration of germinated barley foodstuff: multi-center open trial. Int J Mol Med (2003) 12:701–4. 10.3892/ijmm.12.5.701 14532996

[105] Hanai H Kanauchi O Mitsuyama K Andoh A Takeuchi K Takayuki I Germinated barley foodstuff prolongs remission in patients with ulcerative colitis. Int J Mol Med (2004) 13:643–7. 10.3892/ijmm.13.5.643 15067363

[106] Araki Y Kanauchi O Sugihara H Fujiyama Y Hattori T. Germinated barley foodstuff suppresses dextran sulfate experimental colitis in rats: the role of mast cells. Int J Mol Med (2007) 19:257–62. 10.3892/ijmm.19.2.257 17203199

[107] Scheppach W Sommer H Kirchner T Paganelli G-M Bartram P Christl S Effect of butyrate enemas on the colonic mucosa in distal ulcerative colitis. Gastroenterology (1992) 103:51–6. 10.1016/0016-5085(92)91094-K 1612357

[108] Chung MJ Park JK Park YI Anti-inflammatory effects of low-molecular weight chitosan oligosaccharides in IgE–antigen complex-stimulated RBL-2H3 cells and asthma model mice. Int Immunopharmacol (2012) 12:453–9. 10.1016/j.intimp.2011.12.027 22266066

[109] Verheijden KA Willemsen LE Braber S Leusink-Muis T Delsing DJ Garssen J Dietary galacto-oligosaccharides prevent airway eosinophilia and hyperresponsiveness in a murine house dust mite-induced asthma model. Respir Res (2015) 16:673. 10.1186/s12931-015-0171-0 25849971 PMC4327967

[110] Sagar S Vos AP Morgan ME Garssen J Georgiou NA Boon L The combination of *Bifidobacterium breve* with non-digestible oligosaccharides suppresses airway inflammation in a murine model for chronic asthma. Biochim Biophys Acta (2014) 1842:573–83. 10.1016/j.bbadis.2014.01.005 24440361

[111] Arslanoglu S Moro GE Schmitt J Tandoi L Rizzardi S Boehm G. Early dietary intervention with a mixture of prebiotic oligosaccharides reduces the incidence of allergic manifestations and infections during the first two years of life. J Nutr (2008) 138:1091–5. 10.1093/jn/138.6.1091 18492839

[112] Arslanoglu S Moro GE Boehm G Wienz F Stahl B Bertino E. Early neutral prebiotic oligosaccharide supplementation reduces the incidence of some allergic manifestations in the first 5 years of life. J Biol Regul Homeost Agents (2012) 26:49–59. 10.1088/2058-7058/26/08/11 23158515

[113] Grüber C van Stuijvenberg M Mosca F Moro G Chirico G Braegger CP Reduced occurrence of early atopic dermatitis because of immunoactive prebiotics among low-atopy-risk infants. J Allergy ClinImmunol (2010) 126:791–7. 10.1016/j.jaci.2010.07.022 20832848

[114] Shibata R Kimura M Takahashi H Mikami K Aiba Y Takeda H Clinical effects of kestose, a prebiotic oligosaccharide, on the treatment of atopic dermatitis in infants. Clin Exp Allergy (2009) 39:1397–403. 10.1111/j.1365-2222.2009.03295.x 19508323

[115] van Hoffen E Ruiter B Faber J M’Rabet L Knol EF Stahl B A specific mixture of short-chain galacto-oligosaccharides and long-chain fructo-oligosaccharides induces a beneficial immunoglobulin profile in infants at high risk for allergy. Allergy (2009) 64:484–7. 10.1111/j.1398-9995.2008.01765.x 18507650

[116] Schouten B van Esch BCAM Kormelink TG Moro GE Arslanoglu S Boehm G Non-digestible oligosaccharides reduce immunoglobulin free light-chain concentrations in infants at risk for allergy. Pediatr Allergy Immunol (2011) 22:537–42. 10.1111/j.1399-3038.2010.01132.x 21771085

[117] Xie Q-M Deng J-F Deng Y-M Shao C-S Zhang H Ke C-K. Effects of cryptoporus polysaccharide on rat allergic rhinitis associated with inhibiting eotaxin mRNA expression. J Ethnopharmacol (2006) 107:424–30. 10.1016/j.jep.2006.03.040 16765544

[118] Kong Q Dong S Gao J Jiang C. In vitro fermentation of sulfated polysaccharides from *E. prolifera* and *L. japonica* by human fecal microbiota. Int J Biol Macromol (2016) 91:867–71. 10.1016/j.ijbiomac.2016.06.036 27316763

[119] Trompette A Gollwitzer ES Yadava K Sichelstiel AK Sprenger N Ngom-Bru C Gut microbiota metabolism of dietary fiber influences allergic airway disease and hematopoiesis. Nat Med (2014) 20:159–66. 10.1038/nm.3444 24390308

[120] Ganda Mall J-P Casado-Bedmar M Winberg ME Brummer RJ Schoultz I Keita ÅV A β-glucan-based dietary fiber reduces mast cell-induced hyperpermeability in ileum from patients with Crohn’s disease and control subjects. Inflamm Bowel Dis (2017) 24:166–78. 10.1093/ibd/izx002 29272475 PMC6166688

[121] Halnes I Baines K Berthon B MacDonald-Wicks L Gibson P Wood L. Soluble fibre meal challenge reduces airway inflammation and expression of GPR43 and GPR41 in asthma. Nutrients (2017) 9:57. 10.3390/nu9010057 28075383 PMC5295101

[122] Wang J Wen L Wang Y Chen F. Therapeutic effect of histone deacetylase inhibitor, sodium butyrate, on allergic rhinitis in vivo. DNA Cell Biol (2016) 35:203–8. 10.1089/dna.2015.3037 26859163

[123] Castro-Rodriguez JA Garcia-Marcos L Alfonseda Rojas JD Valverde-Molina J Sanchez-Solis M. Mediterranean diet as a protective factor for wheezing in preschool children. J Pediatr (2008) 152:823–828.e2. 10.1016/j.jpeds.2008.01.003 18492525

[124] Nagel G Weinmayr G Kleiner A Garcia-Marcos L Strachan DP ISAAC Phase Two Study Group. Effect of diet on asthma and allergic sensitisation in the international study on allergies and asthma in childhood (ISAAC) phase two. Thorax (2010) 65:516–22. 10.1136/thx.2009.128256 20522849

[125] Berthon BS Macdonald-Wicks LK Gibson PG Wood LG. Investigation of the association between dietary intake, disease severity and airway inflammation in asthma. Respirology (2013) 18:447–54. 10.1111/resp.12015 23145908

[126] Verheijden KA Digesting the Role of Specific Pre- and Synbiotics in the Prevention of House Dust Mite Asthma [Thesis]. (2015). 1–219.

[127] Kim SY Sim S Park B Kim J-H Choi HG. High-fat and low-carbohydrate diets are associated with allergic rhinitis but not asthma or atopic dermatitis in children. PLoS One (2016) 11:e0150202. 10.1371/journal.pone.0150202 26919190 PMC4769275

[128] Lin Y-P Kao Y-C Pan W-H Yang Y-H Chen Y-C Lee YL. Associations between respiratory diseases and dietary patterns derived by factor analysis and reduced rank regression. Ann Nutr Metab (2016) 68:306–14. 10.1159/000447367 27347884

[129] Miyake Y Sasaki S Ohya Y Miyamoto S Matsunaga I Yoshida T Dietary intake of seaweed and minerals and prevalence of allergic rhinitis in Japanese pregnant females: baseline data from the Osaka Maternal and Child Health Study. Ann Epidemiol (2006) 16:614–21. 10.1016/j.annepidem.2005.11.010 16406247

[130] de Batlle J Garcia-Aymerich J Barraza-Villarreal A Antó JM Romieu I. Mediterranean diet is associated with reduced asthma and rhinitis in Mexican children. Allergy (2008) 63:1310–6. 10.1111/j.1398-9995.2008.01722.x 18782109

[131] Kusunoki T Takeuchi J Morimoto T Sakuma M Yasumi T Nishikomori R Fruit intake reduces the onset of respiratory allergic symptoms in schoolchildren. Pediatr Allergy Immunol (2017) 28:793–800. 10.1111/pai.12817 29024078

[132] Okayama Y Kawakami T. Development, migration, and survival of mast cells. Immunol Res (2006) 34:97–116. 10.1385/IR:34:2:97 16760571 PMC1490026

[133] Dahlin JS Hallgren J. Mast cell progenitors: origin, development and migration to tissues. Mol Immunol (2015) 63:9–17. 10.1016/j.molimm.2014.01.018 24598075

[134] Wang C Shoji H Sato H Nagata S Ohtsuka Y Shimizu T Effects of oral administration of *Bifidobacterium breve* on fecal lactic acid and short-chain fatty acids in low birth weight infants. J Pediatr Gastroenterol Nutr (2007) 44:252–7. 10.1097/01.mpg.0000252184.89922.5f 17255840

[135] Sagar S Demirtekin N Lempsink L Blokhuis BR Redegeld FA Bergenhenegouwen JV Inhibitory effects of beneficial bacteria on recruitment and function of bone marrow cells in a mouse model of chronic allergic asthma. EC Pharmacol Toxicol Editorial (2017) 3(2):26–7.

[136] Asher MI Montefort S Björkstén B Lai CK Strachan DP Weiland SK Worldwide time trends in the prevalence of symptoms of asthma, allergic rhinoconjunctivitis, and eczema in childhood: ISAAC phases one and three repeat multicountry cross-sectional surveys. Lancet (2006) 368:733–43. 10.1016/S0140-6736(06)69283-0 16935684

[137] Kawakami T Ando T Kimura M Wilson BS Kawakami Y. Mast cells in atopic dermatitis. Curr Opin Immunol (2009) 21:666–78. 10.1016/j.coi.2009.09.006 19828304 PMC2839879

[138] Liu F-T Goodarzi H Chen H-Y. IgE, mast cells, and eosinophils in atopic dermatitis. Clin Rev Allergy Immunol (2011) 41:298–310. 10.1007/s12016-011-8252-4 21249468

[139] Barnetson RSC Childhood atopic eczema. BMJ (2002) 324:1376–9. 10.1136/bmj.324.7350.1376 12052810 PMC1123328

[140] Powe DG Groot Kormelink T Sisson M Blokhuis BJ Kramer MF Jones NS Evidence for the involvement of free light chain immunoglobulins in allergic and nonallergic rhinitis. J Allergy Clin Immunol (2010) 125:139–145.e3. 10.1016/j.jaci.2009.07.025 19818484

[141] Schouten B van Esch BCAM van Thuijl AOJ Blokhuis BRJ Groot Kormelink T Hofman GA Contribution of IgE and immunoglobulin free light chain in the allergic reaction to cow’s milk proteins. J Allergy ClinImmunol (2010) 125:1308–14. 10.1016/j.jaci.2010.02.039 20434201

[142] Boženský J Hill M Zelenka R Skýba T. Prebiotics do not influence the severity of atopic dermatitis in infants: a randomised controlled trial. PLoS One (2015) 10:e0142897. 10.1371/journal.pone.0142897 26571488 PMC4646669

[143] Boyle RJ Tang MLK Chiang WC Chua MC Ismail I Nauta A Prebiotic-supplemented partially hydrolysed cow’s milk formula for the prevention of eczema in high-risk infants: a randomized controlled trial. Allergy (2016) 71:701–10. 10.1111/all.12848 27111273 PMC4996326

[144] Kukkonen K Savilahti E Haahtela T Juntunen-Backman K Korpela R Poussa T Probiotics and prebiotic galacto-oligosaccharides in the prevention of allergic diseases: a randomized, double-blind, placebo-controlled trial. J Allergy ClinImmunol (2007) 119:192–8. 10.1016/j.jaci.2006.09.009 17208601

[145] Corrêa-Oliveira R Fachi JL Vieira A Sato FT Vinolo MAR Regulation of immune cell function by short-chain fatty acids. Clin Transl Immunol (2016) 5:e73 10.1038/cti.2016.17 PMC4855267 27195116

[146] Thio CL-P Chi P-Y Lai AC-Y Chang Y-J. Regulation of type 2 innate lymphoid cell-dependent airway hyperreactivity by butyrate. J Allergy Clin Immunol (2018). 10.1016/j.jaci.2018.02.032 29522844

[147] Gri G Frossi B D’Inca F Danelli L Betto E Mion F Mast cell: an emerging partner in immune interaction. Front Immunol (2012) 3:120. 10.3389/fimmu.2012.00120 22654879 PMC3360165

[148] Klose CSN Artis D. Innate lymphoid cells as regulators of immunity, inflammation and tissue homeostasis. Nat Immunol (2016) 17:765–74. 10.1038/ni.3489 27328006

